# Dual anti-idiotypic purification of a novel, native-format biparatopic anti-MET antibody with improved *in vitro* and *in vivo* efficacy

**DOI:** 10.1038/srep31621

**Published:** 2016-08-22

**Authors:** Marie Godar, Virginia Morello, Ava Sadi, Anna Hultberg, Natalie De Jonge, Cristina Basilico, Valérie Hanssens, Michael Saunders, Bart N. Lambrecht, Mohamed El Khattabi, Hans de Haard, Paolo Michieli, Christophe Blanchetot

**Affiliations:** 1 argenx BVBA, Industriepark Zwijnaarde 7, Building C, 9052 Zwijnaarde, Belgium; 2 VIB Inflammation Research Center 9052 Zwijnaarde, Belgium; 3 Department of Internal Medicine, Ghent University, 9000 Ghent, Belgium; 4 Department of Oncology, University of Torino Medical School, 10060 Candiolo, Turin, Italy; 5 Candiolo Cancer Institute, FPO-IRCCS, 10060 Candiolo, Turin, Italy; 6 QVQ BV, Yalelaan 1 Androclus building, 3584 CL Utrecht, The Netherlands; 7 Department of Pulmonary Medicine, ErasmusMC, 3015 GE Rotterdam, The Netherlands

## Abstract

Bispecific antibodies are of great interest due to their ability to simultaneously bind and engage different antigens or epitopes. Nevertheless, it remains a challenge to assemble, produce and/or purify them. Here we present an innovative dual anti-idiotypic purification process, which provides pure bispecific antibodies with native immunoglobulin format. Using this approach, a biparatopic IgG1 antibody targeting two distinct, HGF-competing, non-overlapping epitopes on the extracellular region of the MET receptor, was purified with camelid single-domain antibody fragments that bind specifically to the correct heavy chain/light chain pairings of each arm. The purity and functionality of the anti-MET biparatopic antibody was then confirmed by mass spectrometry and binding experiments, demonstrating its ability to simultaneously target the two epitopes recognized by the parental monoclonal antibodies. The improved MET-inhibitory activity of the biparatopic antibody compared to the parental monoclonal antibodies, was finally corroborated in cell-based assays and more importantly in a tumor xenograft mouse model. In conclusion, this approach is fast and specific, broadly applicable and results in the isolation of a pure, novel and native-format anti-MET biparatopic antibody that shows superior biological activity over the parental monospecific antibodies both *in vitro* and *in vivo*.

Bispecific antibodies (BsAb) represent new emerging antibody (Ab) -based therapeutics. They are generally built with different antigen (Ag)-binding sites (Fab or single-chain Fv), each one used to provide a different target-binding specificity, albeit within the same molecule^1^. This configuration enables a multitude of novel mechanisms of action that cannot be mediated by monospecific antibodies (mAbs), such as targeting of immune cells to tumors^2^. The clinical efficacy and market approval of Blinatumomab (Blincyto^®^, anti-CD19 × anti-CD3)^3^,^4^ and Catumaxomab (Removab^®^, anti-EpCAM × anti-CD3)^5^, supported by over 30 BsAbs in clinical development, including over 13 products in phase II clinical trials or beyond^6^, provide therapeutic validation of the bispecific approach, consequently intensifying efforts to develop optimal BsAb formats. Given the level of accumulated experience with mAbs, the ideal BsAb format for therapeutic use would be an unmodified human immunoglobulin (Ig) G. This format would share stability, pharmacokinetic and other sought-after drug-like properties of therapeutic mAbs, while enabling novel modes of action^1^.

An approach previously tried, was to co-express the two heavy chains (HC) and the two light chains (LC) of two different mAbs in a single host cell. Nevertheless, the random assembly of the four chains resulted in a complex mixture of nine unwanted IgG species (HCs and LCs pairing problems), in addition to the desired BsAb, substantially challenging development from yield, cost and especially purity perspectives^7^. A few BsAbs have been purified from such complex mixtures and advanced into clinical development^8^,^9^, including the clinically approved Catumaxomab^5^. Its production in a single cell line (quadroma) was enabled by preferential species restricted pairing of HCs and LCs of the rat IgG2b and the mouse IgG2a^10^. In addition, purification using protein A was possible as only the desired BsAb and the mouse IgG bind to protein A, and are readily separated by sequential pH elution. However, the high immunogenicity triggered in humans with the mouse and rat components limit such approach clinically, which can only be used in very specific indications for a short period of time^11^.

Nowadays, much more efficient methods for BsAbs production have been developed, facilitating the purification of BsAbs^6^. However, they do have limitations such as having common HCs/LCs, altering the native Ab sequence by adding foreign sequences (linkers to connect Ab fragments), remodelling surfaces (mutations at the interfaces between Fc domains to promote molecular assembly), or engineering novel binding sites into Fc domains^1^. These alterations could have a significant negative impact on expression yield and product stability, as well as an increased potential for provoking anti-drug responses in patients^1^. As a result, manufacturing at scales relevant for the clinic is often a major hurdle for the development of the newer generation of BsAbs^12^,^13^.

Here we describe an innovative approach using a two-step purification process with two specific anti-idiotypic camelid single-domain Ab fragments (VHH) to obtain pure and native-format BsAbs from heterogeneous IgG preparation (Fig. 1). VHH fragments are the smallest intact Ag binding domains derived from heavy-chain-only Abs of camelids (~15 kDa, Supplementary Fig. 1)^14^. The facile identification of Ag-specific VHHs as well as their beneficial biochemical and economic properties (size, affinity, specificity, stability, production cost)^14^ stimulated the use of VHHs as tools in this study.

The main advantage of VHHs for affinity purification is their single domain appearance that facilitates efficient refolding after denaturation. Indeed, Verheesen *et al*. showed that due to extreme refolding capabilities and physical stability of the VHHs, the affinity matrix could be regenerated more than 2,000 times without loss of capacity, while the fragment’s monomeric nature permitted an efficient elution of Ag^15^. By opposition, the HCs and LCs of a conventional Ab and the derived Fab or single-chain Fv fragments have to refold individually, and then associate into a heterodimer molecule for regaining Ag binding capacity. Thus, the physical and thermal stability, or better, the capacity of VHHs to refold completely after denaturation enables the apparent infinite reusability of the columns^15^. These characteristics explained that these binding domains have already been successfully applied as affinity ligands in chromatography processes covering a variety of biotherapeutics^16^,^17^,^18^,^19^, can serve as target-specific capture reagents^20^, and can replace the conventional and expensive protein A purification step^21^,^22^.

To validate this dual anti-idiotypic purification process, we established a proof-of-principle study using two previously generated antagonistic mAbs that compete with HGF for binding to MET, targeting two unique, non-overlapping epitopes on the extracellular region of the MET receptor (Fig. 2a,b)^23^. The first mAb is directed against the PSI-IPT 1 domain (WT46) whereas the second targets the SEMA domain blades 2–3 (WT52) (Fig. 2b). In a previous study, we showed that although cooperation between these two mAbs was reproducibly observed *in vitro* in a variety of biochemical and biological assays, the WT46 and WT52 did not always show a sound synergistic effect in mice^23^. Indeed, cooperation requires both mAbs to be on target at the same time at similar concentrations. This condition can be obtained without difficulty in cultured cells, but in tissues, where Ab concentration depends on a plethora of independent parameters including plasma stability, vessel permeability, drug diffusion, tissue penetration, and protein turnover, the local concentration of two distinct mAbs can vary significantly. Moreover, many environmental factors, including secreted cytokines, extracellular matrix components, and soluble proteases, can influence the exposure of different MET epitopes, preventing equal target engagement by different Abs^23^. This problem can be overcome by the development of a BsAb, also called biparatopic antibody (BpAb) due to its ability to target the two different epitopes of the parental mAbs. This resulting BpAb will most likely possess enhanced avidity compared to the parental mAbs owing to its bivalent paratopic binding. Indeed, it has been demonstrated for a diabody targeting two different epitopes on the extracellular domain of human vascular endothelial growth factor receptor 2, that, by simultaneously binding to two different epitopes on the same target molecule, the BpAb could even potentially acquire new functionality that could not be achieved with the parental mAbs when used alone or in combination^24^.

After active immunization of outbred animals (*Lama glama*) with the parental mAbs, we generated a panel of anti-idiotypic VHHs that specifically bound to these parental mAbs, meaning only able to bind to the properly paired HC/LC and not any of the mispaired combinations (Fig. 1). The two anti-idiotypic VHHs coupled to distinct columns were then sequentially used to purify the BpAb. After confirmation of its purity and functionality, we showed that the inhibitory ability of the anti-MET BpAb was significantly higher than the parental mAbs as assessed in cell-based assays and more importantly in a tumor xenograft mouse model.

## Results

### Generation of specific anti-WT46 and anti-WT52 VHHs

In order to validate the dual anti-idiotypic approach to isolate pure and native-format BsAbs from heterogeneous IgG preparation, two previously generated antagonistic mAbs that compete with HGF for binding to MET were chosen (Fig. 2a)^23^. The first mAb, WT46, is directed against the IPT region (PSI-IPT 1) while the second, WT52, targets the SEMA domain (blades 2–3) (Fig. 2b)^23^. A panel of specific anti-idiotypic VHHs were first generated using llama derived VHH phage display libraries from animals immunized with the llama IgG1 WT46 or WT52 (Supplementary Fig. 2). Indeed, the VH and VL regions of the mAbs were fused to llama constant HC domains from IgG1 and a llama constant LC domain, generating llama mAbs for the immunization. The presence of llama domains ensured that the immune response was focused against the idiotypes, corresponding to the specific complementarity determining regions (CDRs) of the WT46 and WT52, and not against the constant regions of the mAbs.

To select VHHs specific for the CDRs, VHH phage display was performed on the WT46 and WT52 in the presence of an excess of mispaired mAbs (HCs of the WT46 paired with the LCs of the WT52 and the reverse combination) during two rounds of selections. Periplasmic fractions containing VHHs produced by individual clones, were screened by enzyme-linked immunosorbent assay (ELISA) to find two unique anti-idiotypic VHHs. More specifically, after immobilization of the WT46 and WT52, a serial dilution of the anti-idiotypic VHHs (0–1000 nM) was added and the absorbance was measured. Two anti-idiotypic VHHs, specifically recognizing the CDRs of the WT46 or WT52, were found from the panel and named VHH anti-WT46 and VHH anti-WT52, respectively. Specificity was confirmed for the VHH anti-WT46 by showing binding to the WT46 but not to the WT52 (Fig. 3a). Similarly, VHH anti-WT52 bound to the WT52 but not to the WT46 as depicted in Fig. 3b. An EC_50_ of 62 nM was measured for the WT46 binding to the VHH anti-WT46, and of 47 nM for the WT52 targeting the VHH anti-WT52. Moreover, the ability of the anti-idiotypic VHH anti-WT46 and VHH anti-WT52 to compete with their idiotypic mAbs (WT46 and WT52, respectively) for binding to MET, was confirmed by performing a competitive ELISA (Supplementary Fig. 3). Altogether, these data demonstrated that the two anti-idiotype VHHs specifically recognized the CDRs of their targets with affinities allowing their capture as well as their elution^21^.

Thereafter, the VHHs anti-WT46 and anti-WT52 were produced, purified and coupled to sepharose beads. VHH-functionalized sepharose beads were packed into small Tricorn columns and equilibrated before injection of the mAbs. The capacity of the columns was then investigated by injection of the parental mAbs to their respective VHH columns. Both anti-idiotype VHH columns bound all the injected mAbs as depicted in Fig. 3c (WT46) and 3d (WT52). Indeed, no peaks were observed in the first flow-through, indicating that all the mAbs bound to their respective anti-idiotype VHH columns and were eluted after application of the elution buffer.

### Isolation of pure anti-MET biparatopic antibodies using the dual anti-idiotypic purification process

**Figure 1:**
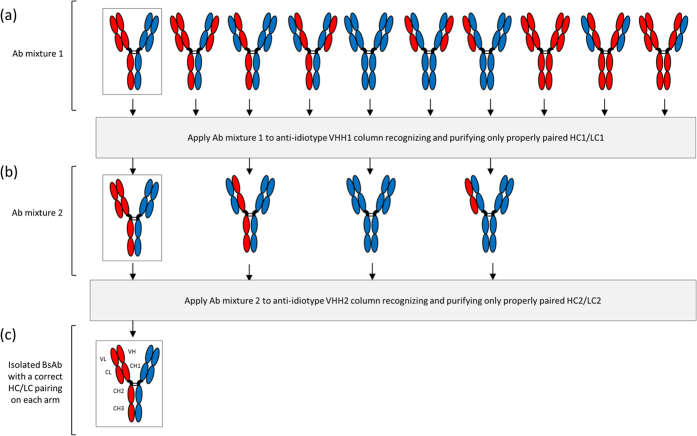
Schematic representation of the dual anti-idiotypic purification process to isolate a desired and properly paired BsAb (framed). (**a**) From a mixture of ten possible combinations formed by alternative LCs and HCs pairings, an anti-idiotypic VHH1 recognizing only the HC1/LC1 pairing is used to extract all four Abs containing this pairing (**b**). Then, a second anti-idiotypic (VHH2) is used to collect the BsAb containing the properly paired HC2/LC2. (**c**) This leads to the isolation of the desired BsAb, which has correct HC1/LC1 and HC2/LC2 pairings.

The anti-MET IgG1 BpAbs were produced by transient transfection of mammalian cells using equal amounts of plasmids encoding the two LCs and the two HCs of both the WT46 and WT52. The Ab mixture resulting from this production was then purified by sequential loading on the VHH anti-WT52 column and the VHH anti-WT46 column (Fig. 1). After washing of the unbound material, the BpAb was eluted from the second column, concentrated, buffer exchanged and filter sterilized. Each step of the purification process was nicely illustrated by the segregation of LCs of two different molecular weights on SDS-PAGE, indicating the presence of the two different mAbs (Fig. 4a). Indeed, varying amounts of the two LCs in the different fractions were observed whereas the eluted fraction from the second column containing the BpAb showed equal amounts of the two LCs. These results suggested that the last eluted fraction from the VHH anti-WT46 column contained pure anti-MET BpAbs.

The purity of the BpAb was then confirmed by using high-resolution mass spectrometry. The purified parental mAbs and the BpAb were analyzed following an on-line desalting step. For each sample the deconvoluted spectra are shown in Fig. 4b. The results illustrated that the BpAb had a molecular mass (148.9 kDa) that was intermediate to those of the parental anti-MET mAbs, WT52 (147.8 kDa) and WT46 (150.0 kDa). These data also confirmed the difference observed on SDS-PAGE for the molecular weight of the LCs of the parental WT46 and WT52 (Fig. 4a).

The presence of BpAbs in the last eluted fraction was further tested by ELISA using human/llama-MET chimeras containing only one of the human epitopes (Supplementary Fig. 4). To this end, MET chimera LP6, which contains the WT52 epitope in the human SEMA domain blades 2–3 and the llama PSI-IPT 1–4 domains, was immobilized before incubation of a serial dilution of the collected fractions (loading, flow-through and elution, 0–67 nM). MET chimera LS5, which consists of the WT46 epitope in the human PSI-IPT 1 domain and the llama SEMA domain, was finally added. As shown in Fig. 4c, MET chimera LS5 was only bound by the last eluted fraction from the VHH anti-WT46 column. As expected, the flow-through fractions of both columns containing mAbs or mispaired Abs showed low or no binding. These data corroborated that the last eluted fraction contained pure anti-MET BpAbs.

Taken together, these results showed that only one Ab population was present in the last eluted fraction confirming the specificity of the anti-idiotype VHHs, which removed all non BsAb species (monospecific or mispaired Abs) and validated further this dual anti-idiotypic approach for the isolation of pure BpAbs.

### Purified anti-MET biparatopic antibody maintains the binding capacities of the parental molecules

The affinity of the BpAb and the parental mAbs for MET or MET chimeras was determined using surface plasmon resonance. As shown in Supplementary Table S1 and Supplementary Fig. 5, the monovalent binding of the BpAb to the MET chimeras LS5 (human PSI-IPT 1 domain, Supplementary Fig. 4) and LP6 (human SEMA domain, Supplementary Fig. 4), resulted in lower affinities compared to the parental mAbs, which benefit of the avid interactions with the MET chimeras. Moreover, no significant difference was observed between the parental mAbs and BpAb when measuring their affinity to MET, demonstrating the avid binding for both the BpAb and the parental mAbs. These results confirmed that both arms of the anti-MET BpAb were functional by maintaining the binding capacities of the parental molecules.

The ability of the purified BpAb to bind simultaneously to the human PSI-IPT 1 domain with one arm and to the human SEMA domain with the other arm, was further confirmed by ELISA. MET chimera LP6 was immobilized and a serial dilution of the BpAb or the parental mAbs (0–133 nM) was applied before addition of the MET chimera LS5 in solution. As depicted in Fig. 5a, only the BpAb could simultaneously bind to the immobilized MET chimera LP6 and to the MET chimera LS5 in solution.

The biparatopic nature of the Ab was also confirmed using surface plasmon resonance. MET chimeras LS5 and LP6 as well as an irrelevant protein (IgG1) were coupled on a CM5 chip on different channels (Fig. 5b). The BpAb was able to bind specifically to MET chimera LP6, and an increased signal was observed on coupled MET chimera LP6 when MET chimera LS5 was then added, indicating that the BpAb was able to simultaneously bind to coated LP6 and to LS5 in solution.

Binding of the BpAb was finally investigated by performing a fluorescence-activated cell sorting (FACS) experiment using the A549 human pulmonary epithelial cell line derived from a lung carcinoma, which expresses physiological levels of MET^25^. A549 cells were incubated with increasing concentrations of the Abs (0–400 nM). Similar binding was observed for the BpAb and the parental mAbs with an EC_50_ of 1.3 nM, 0.8 nM and 1.6 nM for the BpAb, WT46 and WT52, respectively, as depicted in Fig. 5c. These data indicated that the BpAb displayed dose-dependent binding, validating that it recognized membrane-bound MET in its native conformation. Altogether, these results confirmed the specific binding capacities of the purified anti-MET BpAb.

### Purified anti-MET biparatopic antibody displays superior biological activity compared to the parental monoclonal antibodies

The ability of the BpAb to affect HGF-independent MET activity in tumor cells displaying constitutive MET activation was investigated. For this assay, EBC-1 human lung carcinoma cells, which express high levels of MET and contain high basal phosphorylation due to MET gene amplification, were stimulated with increasing concentrations of Abs (0–200 nM)^26^. Using a phospho-MET ELISA assay, we showed that the BpAb inhibited the MET autophosphorylation in EBC-1 cells in a dose-dependent fashion (IC_50_ = 0.4 nM, I_MAX_ = 63%; corresponding to the percent inhibition achieved by the highest Ab dose tested, 200 nM), achieving a stronger effect than the parental WT46 (IC_50_ = 16.4 nM, I_MAX_ = 26%) and WT52 (IC_50_ = 1.5 nM, I_MAX_ = 40%), as shown in Fig. 6a.

The capability of the BpAb to block HGF-induced branching morphogenesis was further analyzed. SV40 T-Ag-transformed LOC human kidney epithelial cell spheroids^27^ were seeded in collagen before stimulation with 1 nM HGF and with increasing concentrations of the parental mAbs (WT46 and WT52) or the BpAb (0–30 nM). The BpAb significantly inhibited HGF-induced branched tubule formation already at a 1:1 molar ratio with recombinant HGF, showing at least three-fold improved inhibition in comparison to the parental mAbs, as depicted in Fig. 6b.

Prompted by the above results, the ability of the BpAb to interfere with HGF-dependent tumor progression was analyzed in a mouse model of human cancer. Since mouse HGF binds to human MET with low affinity and fails to activate it^28^, testing the therapeutic potential of HGF-competing Abs in mice requires a source of human HGF. The most characterized HGF-dependent xenograft model is the U87-MG human glioma cells, which expresses both human MET and HGF^29^. On the basis of previous analysis, the Ab activity was tested at the low dose of 0.5 mg/kg^23^. U87-MG cells were injected subcutaneously into NOD-SCID mice. After 4 weeks, mice were stratified on the basis of tumor volume and divided into four homogeneous groups (*n* = 7), which were randomly assigned to the following treatment, irrelevant IgG1, WT52, WT46 or the BpAb. Abs were administered twice weekly and the tumor growth was followed over time until the study was terminated at day 26 after randomization. This experiment revealed that while the low dose of both the WT46 and WT52 significantly inhibited U87-MG xenograft growth compared to the IgG1 control (*P* values equaled to 0.008 and 0.021, respectively), the BpAb was more effective with complete inhibition of tumor growth until day 15 (*P* value equaled to 0.002) (Fig. 6c). The BpAb was also statistically significant in comparison to the WT46 and WT52 (*P* values equaled to 0.023 and 0.017, respectively). At day 26, the BpAb still showed significant tumor growth inhibition in comparison to the IgG1 control (*P* equals to 0.003), confirming the superior biological activity of the anti-MET BpAb compared to the parental mAbs.

## Discussion

**Figure 2:**
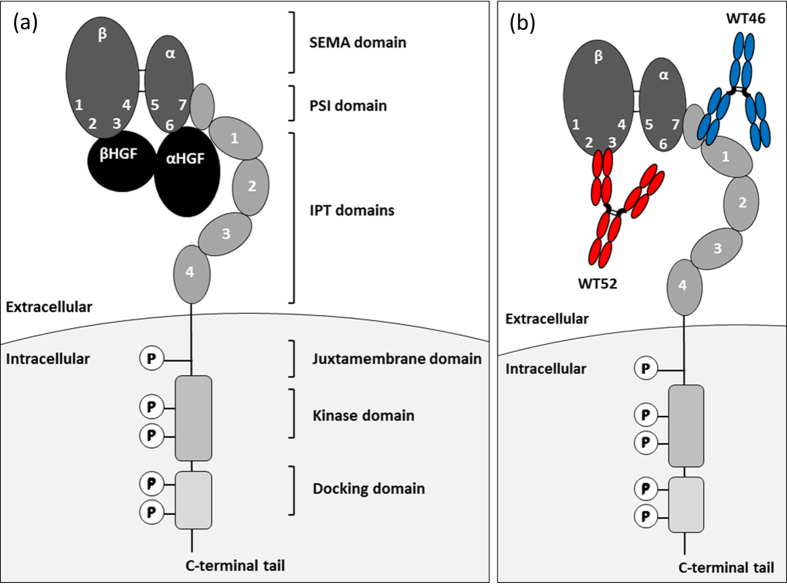
Schematic representation of MET interactions with its natural ligand, HGF, or antagonistic anti-MET mAbs. (**a**) Hypothetical model of HGF/MET interactions. HGF is secreted as a precursor (pro-HGF) that binds to MET at high affinity but does not activate it. Upon proteolytic processing, pro-HGF is converted into a α-β heterodimeric ligand containing a high-affinity MET-binding site in the α-chain, and a low-affinity MET-binding site in the β-chain held together by a disulphide bond. Cooperation between the α- and the β-chains is required for biological activity of HGF; while the α-chain is sufficient for MET binding, the β-chain is necessary for MET activation. MET is a single-pass, multi-domain, disulphide-linked α/β heterodimer. Its extracellular portion consists of three domains. A modular structure encompassing a 7-bladed β-propeller semaphorin homology domain (SEMA), which comprises the whole α-chain and part of the β-subunit; a cysteine-rich plexin-semaphorin-integrin homology domain (PSI), and four immuno-globulin-plexin-transcription factor homology domains (IPT 1–4). The intracellular region consists of the kinase domain and a multifunctional docking site. (**b**) Antagonist anti-MET mAbs that compete with HGF for binding to MET. WT46 (blue) is directed against the PSI-IPT 1 region whereas WT52 (red) is targeting the SEMA domain (blades 2–3).

**Figure 3:**
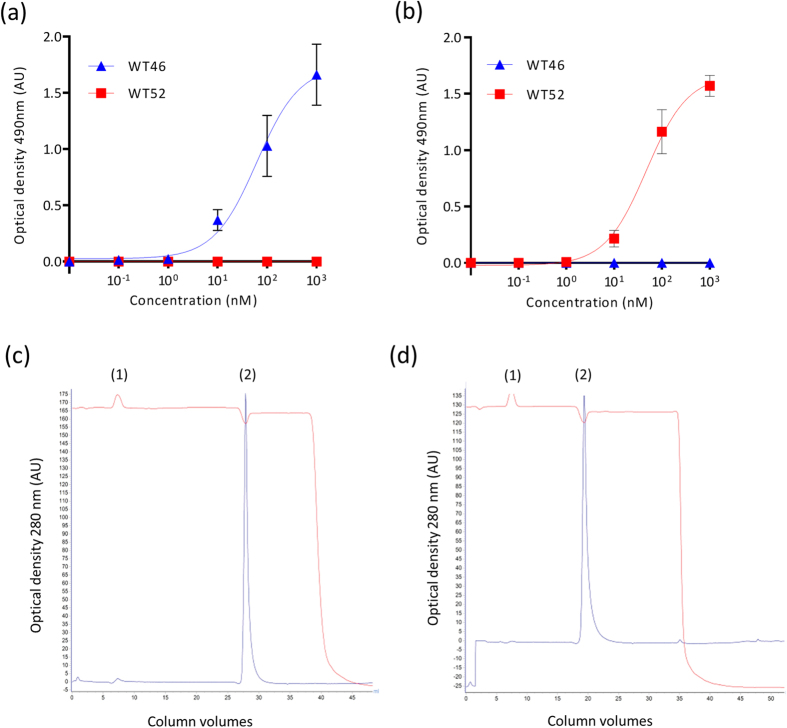
Binding of the VHH anti-WT46 and VHH anti-WT52 to the WT46 and WT52 respectively. ELISA results displaying the specific binding (**a**) of the VHH anti-WT46 to the WT46 (blue triangles) but not to the WT52 (red squares), and (**b**) of the VHH anti-WT52 to the WT52 (red squares) but not to the WT46 (blue triangles). Results are normalized and expressed as mean ± SEM of three independent experiments. Chromatograms validated (**c**) the specific binding (1) and elution (2) of the WT46 to the VHH anti-WT46 column and (**d**) the specific binding (1) and elution (2) of the WT52 to the VHH anti-WT52 column.

**Figure 4:**
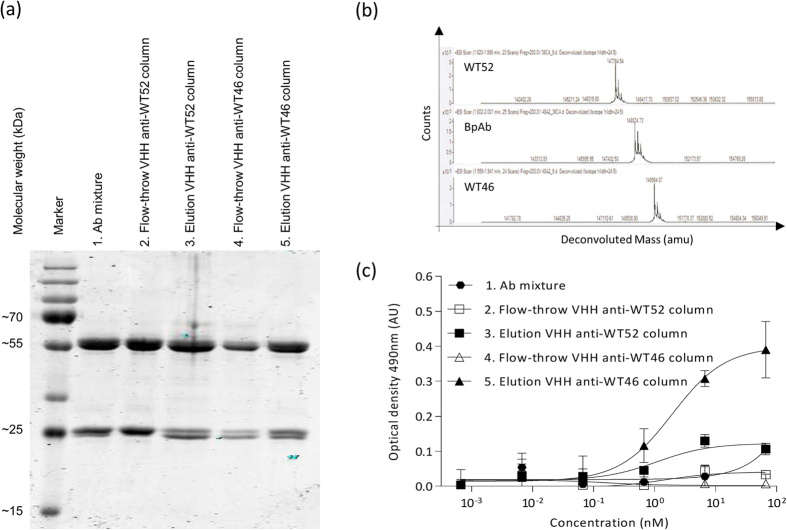
Confirmation of the purity and homogeneity of the BpAb. (**a**) Coomassie staining of SDS-PAGE loaded with the different fractions (loading, flow-through and elution) obtained during the dual anti-idiotypic purification process. The segregation of the LCs of two different molecular weights illustrated the purification process. Indeed, the BpAb eluted from the second column contained equivalent amounts of both LCs (5), whereas the first elution had predominantly the LCs with the lower molecular weight (3) and the initial mixture contained predominantly the LCs with the highest molecular weight (1). (**b**) Mass spectrogram of the anti-MET mAbs and BpAb. BpAb had a molecular mass (148.9 kDa) intermediate to the parental anti-MET mAbs, WT52 (147.8 kDa) and WT46 (150.0 kDa). (**c**) Capture of MET chimera LS5 in solution (containing only the human WT46 epitope) to immobilized MET chimera LP6 (containing only the human WT52 epitope), through a serial dilution of the different fractions collected during the dual anti-idiotypic purification process. MET chimera LS5 was only captured through the last eluted fraction from the VHH anti-WT46 column, corroborated that the last eluted fraction contained anti-MET BpAbs. The Flag Tag on the MET LS5 chimera is detected. Results of the ELISA are normalized and expressed as mean ± SEM of two independent experiments.

**Figure 5:**
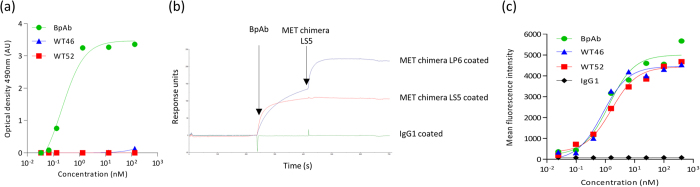
ELISA, surface plasmon resonance and FACS assays confirmed the biparatopic binding abilities of the anti-MET BpAb. (**a**) The pure BpAb (green circles) had the highest titer in capturing the MET chimera LS5 (WT46 epitope) on immobilized MET chimera LP6 (WT52 epitope), in comparison to the parental WT46 (blue triangles) and WT52 (red squares). The Flag Tag on the MET LS5 chimera is detected. Results are normalized and expressed as mean ± SEM of two independent experiments. (**b**) Surface plasmon resonance signals measured after the subsequent injection of the BpAb and the MET chimera LS5 (WT46 epitope), to coated irrelevant IgG1 and MET chimeras LS5 and LP6 (WT52 epitope). (**c**) Binding of the BpAb (green circles), the WT46 (blue triangles), the WT52 (red squares) and an irrelevant IgG1 (black diamonds) to human membrane-bound MET on A549 cells, analyzed by FACS. Bound Ab is proportional to the mean fluorescence intensity.

Anti-idiotypic Abs recognize the CDRs of an Ab and are therefore Ab specific^30^. Otherwise, the facile identification of Ag-specific VHHs as well as their beneficial biochemical and economic properties (size, affinity, stability, production cost)^14^ make them ideal candidates to purify proteins^30^. According to these two observations, we exploited the potential of the camelid anti-idiotypic VHHs to develop an innovative two-step purification process in order to isolate pure and native-format BsAbs from heterogeneous IgG preparation (Fig. 1). To validate this dual anti-idiotypic approach, we established a proof-of-principle study using two previously generated antagonistic mAbs that compete with HGF for binding to MET. The first mAb, WT46, is directed against the PSI-IPT 1 domain, while the second, WT52, targets the SEMA domain blades 2–3 (Fig. 2b). In a previous study, we showed that although cooperation between these two mAbs was reproducibly observed *in vitro* in a variety of biochemical and biological assays, the WT46 and WT52 did not always show a sound synergistic effect in mice^23^. Indeed, cooperation requires both mAbs to be on target at the same time at similar concentrations. Furthermore, combination therapy needs the development and approval of the individual mAbs, involving substantial investment of resources for manufacturing, clinical studies and regulatory review^31^.

During the past decade, dual targeting with BsAbs has emerged as an alternative to combination therapy or use of mixtures. From a technological and regulatory perspective, this makes the development less complex because manufacturing, preclinical and clinical testing is reduced to a single molecule^31^. Therapy with a single dual-targeting drug rather than combinations should also be less complicated for the administration of the treatment^31^. Moreover, BsAb technology has been used as a means to construct novel bivalent Ab molecules with increased avidity for binding, by combining two Abs directed against different epitopes within the same target Ag, resulting in a BpAb^32^,^33^. This increased avidity is likely to be due to the fact that the BpAb, although monovalent to each epitope, is in fact bivalent to its target since both of its binding epitopes are located within the same target molecule.

**Figure 6:**
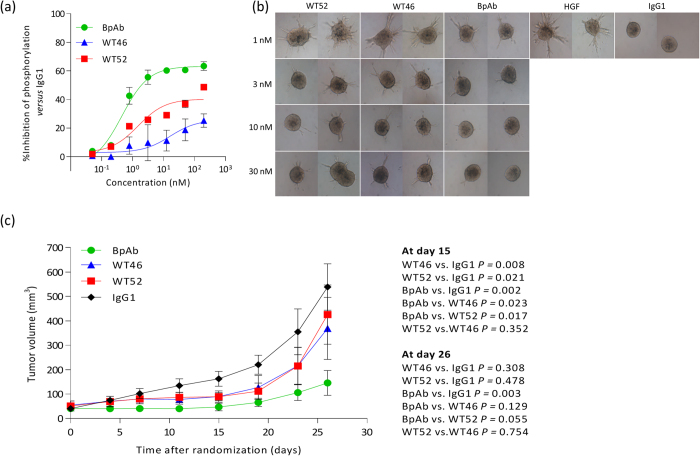
Superior biological activity of the anti-MET BpAb compared to the parental mAbs. (**a**) The BpAb (green circles) inhibited the MET autophosphorylation in EBC-1 cells in a dose-dependent fashion (0–200 nM), achieving a stronger effect than the parental WT46 (blue triangles) and WT52 (red squares). The percentage of phospho-MET levels is compared to cells treated with an irrelevant IgG1. Results are normalized and expressed as mean ± SEM of two independent experiments. (**b**) Superior inhibition of HGF-dependent branching in LOC cell spheroids after 72h of incubation of increasing concentrations of the anti-MET BpAb (0–30 nM) in comparison to the parental mAbs. Branching morphogenesis was assessed by microscopy, magnification x100. (**c**) Improved inhibition of tumor growth in an HGF-dependent human xenograft model (U87-MG human glioma cells, which express both MET and HGF) by the anti-MET Abs (weekly administration of 0.5 mg/kg of WT46, WT52, BpAb or an irrelevant IgG1, *n* = 7). Statistical significance was determined by a Student’s t test. Results are expressed as mean ± SEM.

Here we showed the development of a novel anti-MET IgG1 BpAb, binding to the SEMA domain with one arm (WT52) and to the PSI-IPT domain with the other arm (WT46) (Fig. 2b). The BpAb was purified using anti-idiotypic VHHs that bound specifically to the correct HC/LC pairing of each arm. The high purity was then confirmed by mass spectrometry and functional studies. Binding experiments corroborated that each arm was functional and that the BpAb bound simultaneously to both epitopes on MET molecules. Finally, higher inhibitory abilities of the anti-MET BpAb in comparison to the parental mAbs (WT46 and WT52) were shown *in vitro* using functional cell-based assays and more importantly *in vivo* using a xenograft mouse model, in which the inhibition of the tumor progression was observed after low administered doses of anti-MET Abs (0.5 mg/kg, Fig. 6c). Altogether these data confirmed that blocking simultaneously both HGF binding sites allows superior biological activity over the parental mAbs used alone both *in vitro* and *in vivo.*

One benefit of the dual anti-idiotypic approach is the fact that it does not require any protein engineering at the interface of the HCs/LCs or in the Fc region, suggesting that it can be generalized to any IgG format. Remarkably, the resulting BsAb does not contain mutations and is therefore indistinguishable from a human IgG, conserving its biological activity and eliminating the risk of immunogenicity. Indeed, given the limited number of BsAbs having entered clinical trials, the potential impact on immunogenicity of linkers or mutations introduced at the interface between constant domains that are present in different BsAb formats is currently difficult to evaluate^1^. It is however an important point of consideration that will become better understood, as more BsAbs, using different architectures, will enter in clinical development^34^. It can be expected from the observations made during the last decades with therapeutic mAbs, that ‘foreignness’ of sequence, level of aggregates and purity from contaminants for provocation of anti-drug Abs will also apply to BsAbs^1^.

The disadvantage of the dual anti-idiotypic purification presented here, is that it allows the purification of only a fraction of the total IgG pool produced. Indeed, the use of different chains for the left and the right arms of the Ab generates mixtures, leading to a theoretical yield of 12.5%^7^. More precisely, the two HCs are able to associate in four different combinations, and each of those can associate in a stochastic manner with the LCs, resulting in 10 different Abs of which only one corresponds to the desired functional BsAb^35^. Nevertheless, BsAbs have been successfully generated via this approach where two hybridomas of different specificities were fused together to form a “quadroma” cell line that secretes the two Abs in a mixture that includes the desired BsAb^36^,^37^. Using this technology, Catumaxomab, a BsAb recognizing EpCAM and CD3, was industrially produced and clinically approved in 2009 in the European Union for the intraperitoneal treatment of patients with malignant ascites. Catumaxomab became thereby the first approved BsAb^38^,^10^, demonstrating that the development hurdles of this technique can be overcome^39^,^40^.

Another benefit of the taken strategy is that it appears to be fast after the identification of specific anti-idiotypic VHHs, requiring only a two-step process (no expensive protein A needed). Moreover, the taken strategy is readily scaled up, allowing its applicability in an industrial fashion. As shown, VHHs can be easily coupled to beads, and are stable and resistant to very stringent conditions such as sodium hydroxide treatment required for regeneration and decontamination of the purification columns. Therefore, the physical and thermal stability, or better, the capacity of the VHHs to refold completely after denaturation enables the apparent infinite reusability of the columns^15^.

Since the VHHs are specific to the CDRs, this approach is also compatible to all other BsAb generation approaches previously mentioned. Thus, if in the future these alterations do not show a significant impact on the expression yield and product stability, and do not increase the potential of provoking anti-drug responses in patients, they could easily be combined with our BsAb format and purification method. Finally, the VHH-based purification process is also useful for the BsAbs using these different architectures, because the engineering will principally increase the amount of BsAbs whereas the side products containing incorrectly paired HC-LC combinations or the parental mAbs still need to be removed from the mixtures.

In summary, we used anti-idiotypic VHHs as tools to purify BsAbs with native IgG format. This dual anti-idiotypic purification process was successfully applied to show the improved *in vitro* and *in vivo* efficacy of a novel, pure and native-format anti-MET BpAb in comparison to the potent parental mAbs. More generally, the BsAb format described here represents a flexible approach that provides the means to develop BsAbs with native IgG format, which can readily be produced at large scale for clinical use.

## Methods

### Cell culture

EBC-1, a human lung squamous cell carcinoma line, was purchased from the Japanese Collection of Research Bioresources, and both A549, a human lung carcinoma cell line, and U87-MG, a human glioblastoma cell line, was obtained from the European Collection of Cell Cultures. All cell lines were cultured according to the protocols provided by the supplier. LOC cells have been described previously^27^.

### Antibody production

Antagonist human anti-MET mAbs, WT46 and WT52^23^, were first engineered by combining the llama VH and VL regions to human constant HC domains from IgG1 and a human constant LC domain, respectively generating chimeric llama-human mAbs. The WT46 and WT52 were produced transiently in mammalian cells and purified using Protein A (Thermo Fischer Scientific) as previously described^23^. In parallel, a combination of human mAbs (the HCs of the WT46 paired with the LCs of the WT52 and the reverse combination), were produced as controls for the specificity of the correctly paired human IgG1 BpAb (HC-WT46/LC-WT46_HC-WT52/LC-WT52), mixing each plasmid together (1:1:1:1). The BpAb was produced by co-transfecting 1:1:1:1 of the HCs and LCs of the two human parental mAbs in mammalian cells.

### Immunizations and anti-idiotypic VHH library construction

Two llamas were immunized intramuscularly six times weekly with the llama WT46 and llama WT52 containing the llama constant regions as previously described^23^. Briefly, they received the first two weeks 100 μg of Ags buffered in phosphate-buffered saline (PBS) and mixed with Incomplete Freund’s Adjuvant (Sigma-Aldrich), and 50 μg the remaining four weeks. Five days after the last immunization, 400 mL of immune blood containing peripheral blood lymphocytes was collected from the llamas. They were purified by centrifugation on a Ficoll-Paque gradient and used for extraction of total RNA. Total RNA was then converted into random primed cDNA using reverse transcriptase, and gene sequences encoding for the variable domain of VHHs were isolated as described in the literature^41^.

### Phage display for selection of anti-idiotypic VHHs

Selections from the VHHs containing phage libraries were done as previously described^42^. Briefly, VHH libraries of a size superior at 10^7^ were used. Immobilized mAbs with the human IgG1 constant domains (WT46 or WT52) at the concentrations of 5, 0.5 and 0.05 μg/mL were chosen for the first round and phages were added to the wells containing a 10-fold excess of mispaired Abs compared to the immobilized amounts. For the second round of selection 2, 0.2 and 0.02 μg/mL were immobilized and phages were added in the presence of 20 μg/mL of mispaired Abs. After two rounds of selection, a significant enrichment (superior than 100 fold) was obtained over non-coated wells and 96 VHHs were screened for specificity on ELISA. Over 90% positive hits were found and the positive VHHs were sequenced. Phage productions were performed according to standard protocols and VHHs with His_6_ -tags were then produced in periplasmic fractions from the individual VHHs as previously described^43^.

### VHH ELISA screening

After overnight immobilization at 4 °C with the WT46 or WT52 (2 μg/mL) onto maxisorb plates (Thermo Scientific), the wells were washed with PBS (0.005% v/v) -Tween-80 and blocked with milk protein in PBS (4% w/v, Marvel). A serial dilution of the VHHs anti-WT46 and anti-WT52 was then performed (0–1000 nM) and incubates 1h at room temperature before addition of an anti-Flag Tag Ab (M2, 1/5000, Sigma-Aldrich) 1h at room temperature. After a washing step, a secondary donkey anti-mouse Ab coupled to a horseradish peroxidase (1/5000, Jackson ImmunoResearch) was incubated 1h at room temperature. After a last washing step, o-Phenylenediamine dihydrochloride substrate was added (1/1000, Sigma-Aldrich), the reaction was stopped with 1 M sulfuric acid and the optical density was measured at 490 nm.

### Recloning, production and purification of VHHs

Positive VHHs were cloned into an expression vector pMEK222 (derived from phagemid vector pUR8100^43^, containing Flag and His_6_ tags, but no gene 3). After transformation into *E. coli* strain TG1 and overnight culture in the presence of glucose (2% v/v) and ampicillin (0.1% v/v), expression of the VHHs was induced by 1 mM isopropyl β-D-1-thiogalactopyranoside (Sigma-Aldrich) under low glucose concentrations (0.1% w/v). The produced VHHs were then purified via the His_6_ tag using TALON his-tag purification resin according to standard protocol (Clontech).

### VHH affinity purification columns

10.0 mg VHHs anti-WT52 and 8.4 mg VHHs anti-WT46 were then coupled to 1 mL NHS-sepharose beads according to the manufacturer’s protocol (GE Healthcare). VHH-functionalized sepharose beads were packed into small Tricorn columns (GE Healthcare), one for each anti-idiotypic VHH. Analysis of the specificity of the columns for the parental mAbs was studied using chromatography (AKTA prime, GE Healthcare). More specifically, VHH columns were equilibrated in PBS and mAbs were injected in PBS on the columns. After washing of the unbound mAbs, bound mAbs were eluted with 20 mM citrate buffer, 150 mM sodium chloride pH 3.0, and subsequently neutralized with 1 M potassium phosphate pH 8.0 (1/10 of the eluted volume). The AKTA, controlled by the UNICORN^TM^ software (GE Healthcare), and columns containing the VHHs were then treated with 0.1 M sodium hydroxide and then extensively washed with LAL reagent water (Lonza).

### Dual anti-idiotypic purification process

Ab mixture was first injected into the VHH anti-WT52-functionalized column before injection of the first eluted fraction into the VHH anti-WT46-functionalized column. Unbound material was collected whereas bound Abs were eluted with 20 mM citrate buffer, 150 mM sodium chloride pH3.0 and subsequently neutralized with 1 M potassium phosphate pH 8.0 (1/10 of the eluted volume). Finally, the BpAb was concentrated, buffer exchanged to PBS (0.02% v/v)-Tween-80 and filter sterilized. The purity of the eluted BpAb was tested by measurement of absorption at 280 nm, SDS-PAGE (2 μg of each sample) and high-resolution mass spectrometry (Research Institute for Chromatography, Kortrijk, Belgium).

### Anti-MET biparatopic ELISA

Two similar ELISAs were performed to detect the anti-MET BpAb. The first one was set-up to identify the BpAb in the different fractions (loading, flow-through and elution) obtained during the successive dual anti-idiotypic purification process, and the second one to confirm both the presence of the BpAb in the last eluted fraction and the absence of the parental mAbs. MET chimera LP6, which contains the WT52 epitope in the human SEMA domain blades 2–3 (Supplementary Fig. 4, described in reference^23^), was immobilized (2 μg/mL) in PBS overnight at 4 °C onto maxisorb plates (Thermo Scientific). After a blocking step with milk protein in PBS (4% w/v, Marvel), incubation of a dilution series of the IgGs present in the different collected fractions (0–67 nM) for the first assay, or of the BpAb and parental mAbs (0–133 nM) for the second assay was performed. Subsequently, Flag tagged MET chimera LS5, which contains the WT46 epitope in the human PSI-IPT 1 region (Supplementary Fig. 4) was added (1 μg/mL). Bound MET chimera LS5 was then detected with a mouse anti-Flag tag Ab (M2, 1/5000, Sigma-Aldrich) and a secondary donkey anti-mouse Ab coupled to a horseradish peroxidase (1/5000, Jackson ImmunoResearch). After a last washing step, o-Phenylenediamine dihydrochloride substrate (1/1000, Sigma-Aldrich) was added, the reaction was stopped with 1 M sulfuric acid and the optical density was read at 490 nm.

### Mass spectrometry

Data were acquired using an Agilent Technologies 1290 UHPLC system hyphenated to an Agilent Technologies 6540 Q-TOF equipped with a Jetstream ESI source. Samples were desalted on-line using a reversed-phase cartridge prior to introduction into the MS system. The Q-TOF system was calibrated in the Extended Dynamic Range mode (2 GHz mode). An amount of 4 μg was loaded onto the column. Generated spectra were deconvoluted using a maximum entropy algorithm thereby converting m/z data into molecular weight information.

### Surface plasmon resonance

The BpAb (40 μg/mL) was injected onto a CM-5 chip (GE Healthcare) coupled with the MET chimera LP6 (500 RU) in sodium acetate buffer pH 4.5 at a flow rate of 30 μL/min. After injection of the BpAb, MET chimera LS5 (40 μg/mL) was injected and captured by the BpAb at saturating concentrations. Evaluation of the results was performed using the BIAEval software (GE Healthcare). An irrelevant IgG1 was used as negative control.

### FACS assay

Binding of the BpAb and the parental mAbs to A549 human lung carcinoma cells was analyzed by flow cytometry, as previously described^44^. Briefly, A549 cells were incubated for 30 minutes with increasing concentrations of Abs (0–400 nM). Binding was revealed using phycoerythrin-conjugated anti–human IgG1 Ab (1/200, eBioscience). An irrelevant IgG1 was used as negative control.

### HGF-dependent and -independent *in vitro* assay

The BpAb-mediated inhibition of HGF-independent MET phosphorylation in EBC-1 cells was performed as described for MKN-45 cells^28^. Concisely, the cells were incubated 24 hours in the absence of serum and in the presence of increasing concentrations of Abs (0–200 nM). MET autophosphorylation was determined by ELISA using phospho-MET-specific Abs (1/5000, Sigma-Aldrich). Otherwise, the BpAb mediated inhibition of HGF-induced branching morphogenesis in SV40 T-Ag-transformed LOC human kidney epithelial cell spheroids was performed as previously described^28^. Briefly, SV40 T-Ag-transformed LOC cells were seeded in collagen and then stimulated with 1 nM HGF and increasing concentrations of the parental WT46 and WT52 or the BpAb (0–30 nM). Branching was determined by microscopy 72 hours later. An irrelevant IgG1 was used negative control (1 nM).

### *In vivo* subcutaneous U87-MG xenograft model

Animal studies with mice were conducted in accordance with local (Turin) and European guidelines after ethical approval. The mice were housed in sterilized filter topped cages and maintained under sterile conditions. Food and water were provided *ad libitum*. Six to eight weeks old NOD-SCID mice (Charles River) were injected subcutaneously with 3 × 10^6^ U87-MG cells, expressing MET and autocrine HGF, into the right or left hind flank. When the tumors approached a size of 100 mm^3^, the mice were stratified, divided into groups and treatment with irrelevant IgG1, the WT46, the WT52 and the BpAb, started. The experiment was performed with a dose of 0.5 mg/kg (*n* = 7/group), intraperitoneally injected twice per week until termination of the experiment at day 26 after the stratification of the groups. Upon treatment, the tumors were measured using a caliper and the tumor volume was calculated using the formula V = 4/3π × (1/2*x*) × (1/2*y*) × (1/2*z*), where *x*, *y* and *z* are the three dimensions of the tumor. Mice underwent terminal pentobarbital anesthesia before euthanasia or upon termination of the experiment.

### Statistical analysis

Graphs were compiled and statistical analyses were performed using GraphPad Prism 6.01 software. In all figures, values are expressed as mean and error bars represent SEM. Statistical significance was determined using a 2-tailed homoscedastic Student’s test for the *in vivo* experiments. A *P* value of less than 0.05 was considered statistically significant.

## Additional Information

**How to cite this article**: Godar, M. *et al*. Dual anti-idiotypic purification of a novel, native-format biparatopic anti-MET antibody with improved *in vitro* and *in vivo* efficacy. *Sci. Rep.* **6**, 31621; doi: 10.1038/srep31621 (2016).

## Supplementary Material

Supplementary Information
