# Supplementary material for: Dual anti-idiotypic purification of a novel, native-format biparatopic anti-MET antibody with improved in vitro and in vivo efficacy
Source: Sci Rep. 2016 Aug 22;6:31621. doi: 10.1038/srep31621 (PMC4992859; doi:10.1038/srep31621)
Supplement: Supplementary Information [file srep31621-s1.doc]

**Dual anti-idiotypic purification of a novel, native-format biparatopic anti-MET antibody with improved *in vitro* and *in vivo* efficacy**

**Marie Godar^1,2,3^, Virginia Morello^4,5^, Ava Sadi^6^, Anna Hultberg^1^, Natalie De Jonge^1^, Cristina Basilico^5^, Valérie Hanssens^1^, Michael Saunders^1^, Bart N. Lambrecht^2,3,7^, Mohamed El Khattabi^6^, Hans de Haard^1^, Paolo Michieli^4,5^ & Christophe Blanchetot^1^**

**
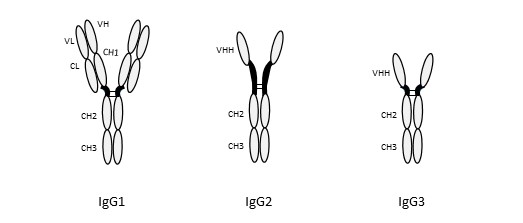
**

**Supplementary Figure 1**. Schematic representation of naturally occurring Abs in sera of camelids. The conventional Abs (IgG1) contain two LCs (VL and CL domains) and two HCs (composed of the VH, CH1, hinge, CH2 and CH3 domains) whereas the two types of homodimeric heavy-chain Abs, IgG2 and IgG3, comprise only HCs; each HC contains a VHH, hinge, CH2 and CH3 domains. The hinge of the IgG2 fraction is longer than the one of the IgG3 type. The smallest intact functional antigen-binding fragment of heavy-chain antibodies is the single-domain VHH.


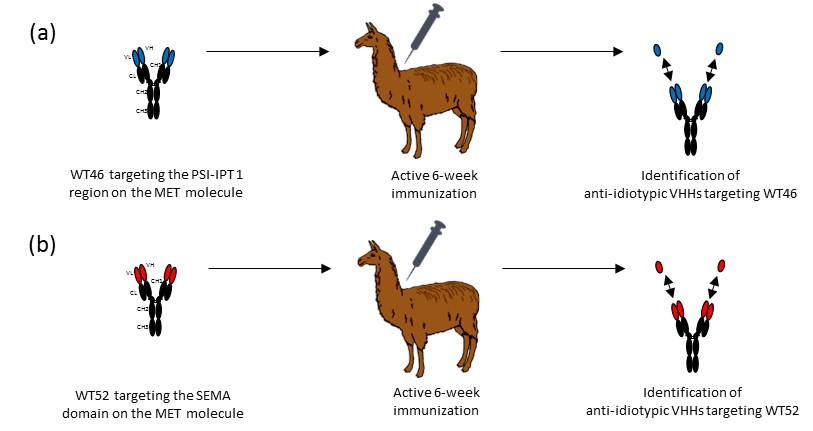


**Supplementary Figure 2.** Schematic representation of the generation of camelid-derived anti-idiotypic VHHs. The VH and VL regions of the human parental mAbs (WT46 and WT52, depicted in blue and red, respectively) were fused to llama constant heavy chain domains from IgG1 and a llama constant light chain domain (shown in black), generating chimeric llama-human mAbs. (**a**) The anti-idiotypic anti-WT46 VHHs were obtained by immunizing a llama with the chimeric llama-human WT46 comprising the first antigen-binding site of the BpAb directed against the PSI-IPT 1 region. (**b**) The anti-idiotypic anti-WT52 VHHs targeting WT52 were got by immunizing a llama with the chimeric llama-human WT52 comprising the second antigen binding site of the BpAb and targeting the SEMA domain (blades 2–3).


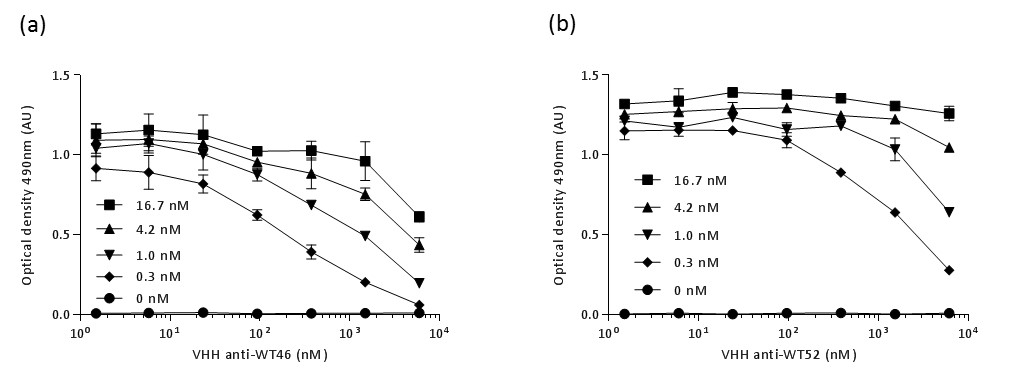


**Supplementary Figure 3.** Confirmation of the ability of the anti-idiotypic VHHs to compete the interaction between the idiotypic mAbs and MET, determined by competitive ELISA. (**a**) The VHH anti-WT46 (0-6000 nM) competed with its idiotypic mAb, WT46 (0-16.7 nM), for binding to coated MET (**b**). The VHH anti-WT52 (0-6000 nM) competed with its idiotypic mAb, WT52 (0-16.7 nM), for binding to coated MET. Results are expressed as mean ± SEM of two independent experiments.


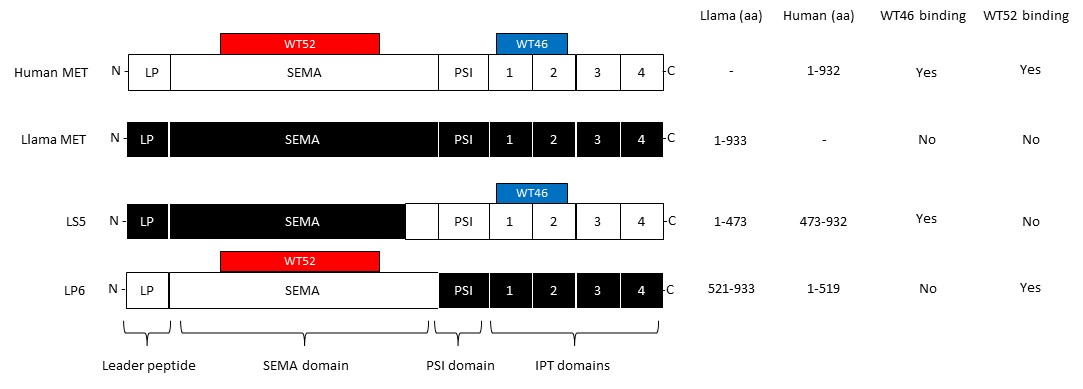


**Supplementary Figure 4.** Schematic representation of the llama-human chimeric MET proteins. The extracellular portions of llama MET and human MET are composed of 933 and 932 amino acids (aa), respectively (llama MET contains an insertion after aa 166). Both receptor ectodomains comprise a leader peptide, a semaphorin homology domain (SEMA), a plexin semaphorin-integrin homology domain (PSI) and four immuno-globulin-transcription factor-plexin homology domains (IPT 1-4). Met chimera LS5 has an N-terminal llama portion followed by a C-terminal human portion. Met chimera LP6 has an N-terminal human portion followed by a C-terminal llama portion. The presence of the epitopes for the WT46 (blue) and WT52 (red) is indicated by a box above.

**
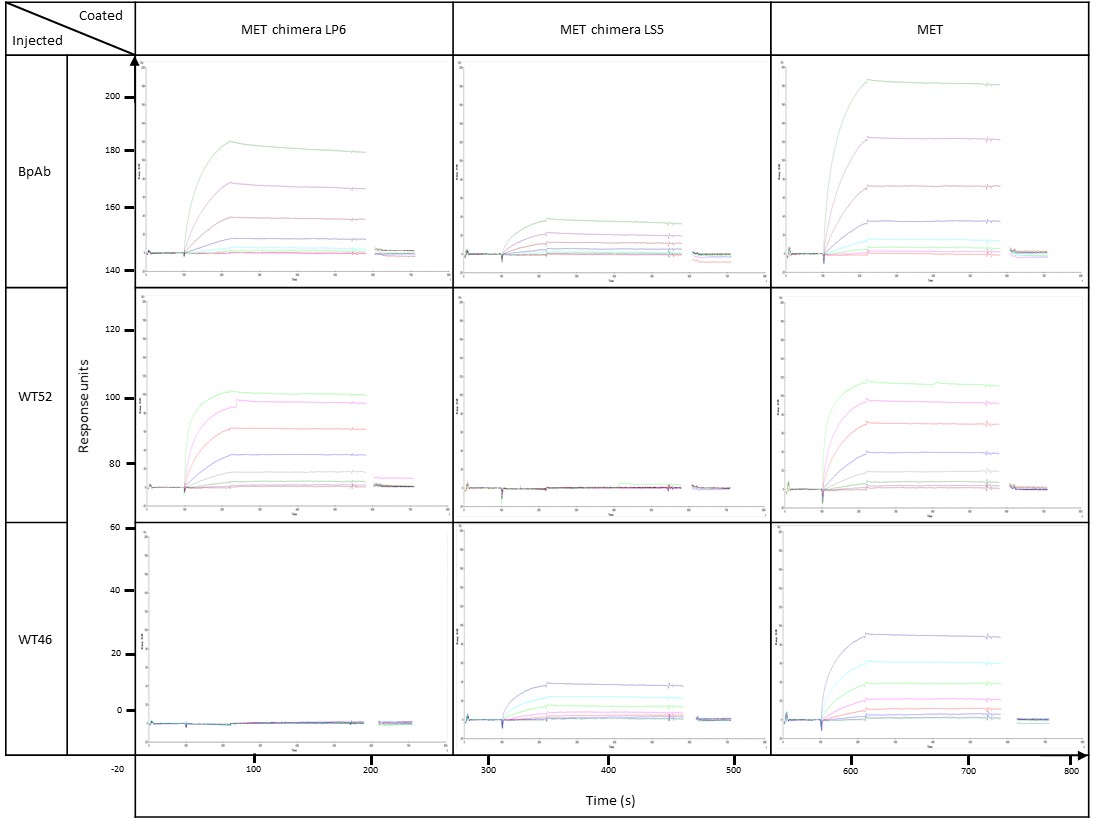
**

**Supplementary Figure 5.** Representative surface plasmon resonance sensorgrams of the interactions between the BpAb or the parental mAbs (WT46 and WT52) at varying concentrations (0-80 µg/mL), and immobilized proteins (MET or MET chimeras).

**Supplementary Table S1.** Determination of the affinity values for the interactions between the BpAb or the parental mAbs (WT46 and WT52), and MET or MET chimeras using surface plasmon resonance.
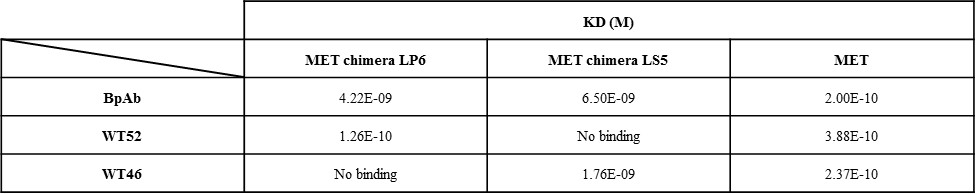


BpAb binds to the MET SEMA domain with one arm (parental WT52) and to the PSI-IPT domain of the MET molecule with the other arm (parental WT46). MET chimera LP6 contains the WT52 epitope in the human SEMA domain blades 2-3 and the llama PSI-IPT 1-4 domains. MET chimera LS5, consists of the WT46 epitope in the human PSI-IPT 1 domain and the llama SEMA domain. A serial dilution (0-80 µg/mL) of the BpAb and the parental mAbs, WT46 and WT52, were injected at a flow rate of 30 µL/min onto a CM-5 chip coupled with the MET chimera LP6 (500 RU), MET chimera LS5 (500 RU) or MET (500 RU). The affinity values (K_D_) are expressed in M. Kinetic analyses were performed with BiaEval Software.
